# Quasioptics for increasing the beam efficiency of wireless power transfer systems

**DOI:** 10.1038/s41598-022-25251-w

**Published:** 2022-12-03

**Authors:** Ricardo A. M. Pereira, Nuno Borges Carvalho

**Affiliations:** Institute of Telecommunications, University of Aveiro, Aveiro, Portugal

**Keywords:** Engineering, Electrical and electronic engineering, Micro-optics, Transformation optics, Renewable energy, Energy infrastructure, Power distribution

## Abstract

The highest beam efficiency in a wireless power transfer (WPT) system that uses focusing components was 51%, using a $\approx {3}\,{\textrm{m}}$ diameter reflector for a transfer distance of ${7.62}\,{\textrm{m}}$. We have beaten that record, and present here a system that surpasses it by 25%. Using the quasioptical framework for reducing spillover losses in WPT, we present a double-reflector system that achieved a higher beam efficiency than the state-of-the-art. The transmitting and receiving antennas were 3D-printed conical smooth-walled horn antennas, specially designed for this purpose. The theoretical analysis enabled the design of a ${5}\,{\textrm{m}}$ system, whose energy focus location has been experimentally verified. Then, the complete system was experimented upon, enabling a high beam transfer efficiency of 63.75%. Additionally, the advantage of using quasioptics in radiative wireless power transfer applications is discussed, as well as the sensitivity of its systems. Finally, a comparison with the state-of-the-art is done by the proposal of new figures-of-merit, relating the systems’ physical dimensions and beam efficiency. This research is a paradigm shift by presenting a promising path for future WPT research through quasioptics, whose high efficiencies may enable commercial applications of this technology for solving power supply issues in our society.

## Introduction

The first wireless power transfer (WPT) experiment was done by Heinrich Hertz, but it was Nikola Tesla who recognized its potential for wirelessly powering devices and actively pursued this goal^1,2^.

To achieve high efficiency wireless power transfer, electromagnetic (EM) radiation can be focused into a beam and directed towards intended targets. This is more easily achieved at higher frequencies, since the system dimensions can be reduced, which only became technologically possible in the 1960s, in great part due to the developments by William Brown in the U.S.^3–6^.

Brown experimented with a wirelessly-powered helicopter^7,8^, developing the rectenna (antenna and rectifying circuit) for it. This enabled two important experiments that still hold WPT records: one for the maximum DC-DC efficiency of 54%^9^ and the other for the highest outputted DC power of ${30.4}\,{\textrm{kW}}$ for an impressive distance of ${1540}\,{\textrm{m}}$ (1 mile)^10^.

The work of Brown which achieved the absolute highest beam efficiency is also Ref.^9^, but the purpose of this project was to study the DC-RF conversion of rectennas. For this reason, no focusing components were used and the transmitting horn antenna was positioned close to the receiver, at merely ${1.7}\,{\textrm{m}}$. The rectenna was designed and built with a sufficiently large area so that the majority of the beam is captured by it. However, the lack of focusing components means that increasing the distance also increases the spillover losses as the radiation divergence becomes significant. On the other hand, systems with focusing components achieve not just higher distances but also maintain higher efficiencies through a range of distances. For these reasons, systems with focusing components must be differentiated. In this category, the record beam efficiency is 51% for a distance of ${7.62}\,{\textrm{m}}$ using a reflector with $\approx {3}\,{\textrm{m}}$, achieved also by Brown^8^. This is the project which we will compare our work with.

Naturally, interest in the field prompted WPT projects all around the world, from Europe^11,12^ to Japan^13–15^, among many others. Most dealt with important aspects of microwave generation or rectification, with few explicitly discussing the beam efficiency. This interest arises from WPT’s potential applications, especially where the installation of traditional power supply lines is not feasible or even possible: ground-to-ground WPT to supply power to hard-to-reach locations, ground-to-air for powering airborne platforms for surveillance and communication purposes^16,17^, or even space applications, such as the generation of solar power in outer space to transmit it back down to Earth via microwaves. The latter is widely known as the Solar Power Satellite (SPS) project^18,19^.

However, the beam divergence is still a considerable problem and the difficulty in achieving highly efficient WPT systems has impeded their implementation in real-life situations. Hence, this project aims to understand and control microwave beams and the way they propagate, for maximizing the beam efficiency, which may then be used in any radiative WPT application. This is done by the implementation of the quasioptical (QO) framework^20^. This theory adapts the tools of optics to contexts with high divergence and can therefore be used to study microwaves traveling distances of meters and kilometers.

The use of quasioptics is paramount for reducing spillover losses, since the beam radius is a known quantity, which can be controlled. Nevertheless, its implementation in WPT has been mostly theoretical and only seldom have experimental systems been developed. As far as we are aware, no complete WPT system has been analyzed through it.

The first system we studied and developed through QO is composed of two sets of a conical horn and parabolic reflector, that were analyzed theoretically using the reciprocity principle. This paper reports the project from theory to complete system experimentation.

**Figure 1:**
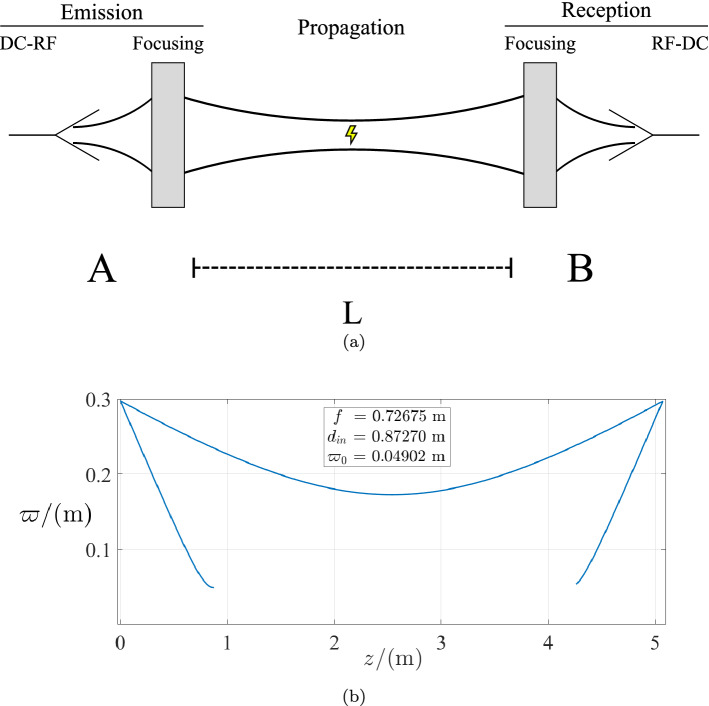
(**a**) Generic quasioptical wireless power transfer system overview. (**b**) Representation of the gaussian beam radius throughout the complete system.

The experiment most similar to this work consists of a horn-to-horn power transfer system^21^, performed at terahertz frequencies, using elliptical mirrors. Maximum beam efficiency of $\approx$ 60% was obtained for a distance of ${13.6}\,{\textrm{cm}}$ at a power of under ${8}\,{{\upmu }\textrm{W}}$. Although the quasioptics analysis is implemented, the system transfer distance is not related to the components’ parameters, as is here.

Finally, the summary in Table 1 serves as a benchmark with other experimental projects whose beam efficiencies are given explicitly or are possible to calculate, to serve as comparison points to this work.

**Table 1:** Wireless power transfer state-of-the-art.

Year	Team	L^1^	Freq.	#^2^	D^3^	FOM	$\eta _{Beam}$	FOM	FOM	Ref.
		(m)	(GHz)		(m)	Dim.	(%)	Beam$_1$	Beam$_2$	
1964	W. Brown	7.62	2.45	1	2.89	17.38	50.9	14.46	8.87	^8^
1974	W. Brown^4^	1.7	2.45	0	1.3	9.58	94	9.31	9.01	^9^
1975	W. Brown^5^	1540	2.388	1	26	53.84	11.3	44.37	6.08	^10^
2011	Gonzalez^6^	0.136	799-938	2	0.03	26.14	60	23.92	15.69	^21^
2016	Gowda	0.4	5.8	0	0.217	8.53	33.2	3.74	2.83	^30^
2022	Pereira^7^	5	5.8	2	1.06	20.57	63.75	18.68	13.32	N.A.

The beam efficiency is as defined in Eq. (5), including all the components except the microwave generation, rectification and the cable losses. Finally, the figures of merits (FOM) are described in Eqs. (1), (2), and (3).

^1^Power transfer distance.

^2^Number of focusing components.

^3^Largest component dimension.

^4^Highest DC-DC efficiency.

^5^Highest outputted DC power.

^6^WPT in the Fresnel zone.

^7^This work, quasioptical WPT.

The main novelty of this work is therefore the implementation of the quasioptics theory to wireless power transfer systems in order to increase the beam efficiency. As far as we are aware, this study contributes to the state-of-the-art (SOA) in several ways: firstly, a record beam efficiency has been achieved in WPT systems; secondly, this claim can be verified by new figures of merit which are here proposed; on the other hand, no QO analysis has been applied to complete WPT systems, relating the gaussian beam’s parameters with the systems components, as is the case here. Finally, the QO analysis of this double-reflector system has not been done before, with the obtained experimental results validating the theoretical ones.

## Increasing the beam efficiency through quasioptics

This work aims to transfer power wirelessly by directing microwave beams towards an intended target. To create this beam, the quasioptics framework is implemented. Hence, this project follows the works that focus electromagnetic radiation for creating a highly directed beam, such as the work of William Brown, and we are therefore working on the Fresnel zone. From this outset, some characteristics can already be assumed: by being directive, the receiver is limited to a certain area. Instead of the power being collected by several devices, we have specified a target device.

This approach requires adding focusing components which have the disadvantages of added cost, complexity and components’ loss. Nevertheless, their implementation significantly reduces the spillover losses, resulting in an overall higher beam efficiency.

The implementation of focusing components can be further optimized by using the quasioptical framework, which consists in the careful design of all radiative components and their precise positioning and alignment. Besides the additional complexity, there is no disadvantage to using quasioptics in systems that contain focusing components. This tool is useful for analyzing radiation beams with considerable divergence, enabling us to understand and control beams throughout the entire system. Here, these diverging beams are microwaves travelling distances of meters. Contrarily to optics, the radiation is no longer represented by thin rays but by a beam with a certain gaussian power distribution^20^. For an engineer, implementing quasioptics may be more complex but not necessarily harder: it involves more design steps but these are compatible with fast system design, prototyping and experimentation.

In practice, there are two main advantages to implementing this approach: QO provides information about the beam radius (Eq. 12) which, if it is maintained smaller than the system components, effectively reduces the spillover losses. On the other hand, how well the antennas generate and receive gaussian beams is accounted for through the coupling efficiency (Eq. 11), which should be optimized for increasing the beam efficiency. Neither of these can be optimized without quasioptics.

The QO system that was first conceptualized is represented in Fig. 1a. In simple terms, the EM energy is transferred from point A to point B, traversing a distance of *L*, while using focusing elements for optimizing the beam, controlling its divergence. For simplicity, the reciprocity principle was applied, making the components in A the same as those in B, but in reverse order.

This simple concept developed into a double-reflector system (Fig. 1b) that was theoretically analyzed^22^, whose main results are the relationships between the reflectors’ *focal length*, *f*, and the wireless power *transfer distance*, *L* (Eq. 14). Following these results, the actual system needed to be defined, after which it was built and subject to experimentation: two horn antennas were designed and manufactured and parabolic reflectors with specific focal lengths were obtained; using only one reflector, the focus location was measured experimentally^23^, validating the system for further experimentation.

It is worth noting that the frequency of operation is a parameter of the gaussian beam and as expected the system design and overall efficiency depend on it. Nevertheless, the theory can be implemented in other frequencies as we have shown in another QO system designed for ${24}\,{\textrm{GHz}}$ using a dielectric lens^24^.

On the other hand, it is important to note that the microwave beam generation and reception were done by a portable network analyzer, hence, these efficiencies are not considered. The main goal was to study the beam transfer and only its efficiency is discussed here, which includes all WPT components except the microwave excitation and reception (Eq. (4) and (5)). This is also referred to in the literature as “RF-RF” efficiency. Since the creation of a beam through quasioptics is the fundamental aspect of this work, “beam” efficiency will be used throughout this paper.

**Figure 2:**
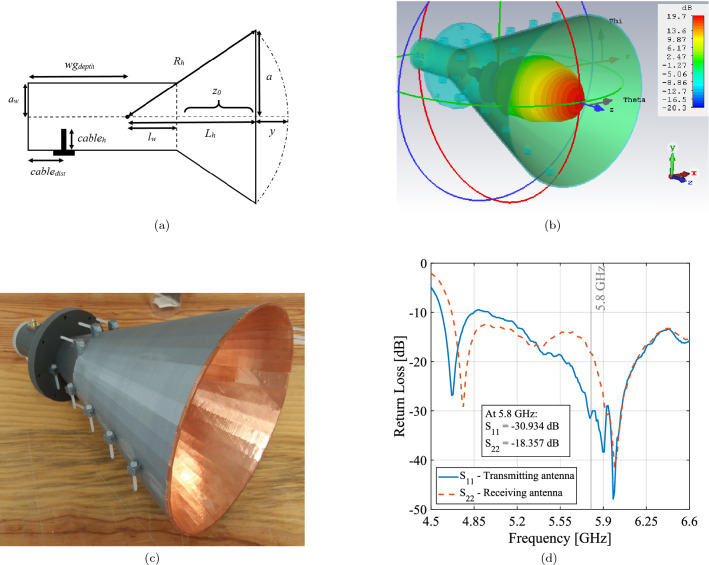
(**a**) Conical horn antenna schematic where *a* is the aperture radius, $L_h$ is the length between the aperture and the center of curvature and $R_h$ is the horn slant length. (**b**) Horn far field simulation result. (**c**) 3D-printed horn antenna. (**d**) Both horn antennas’ return losses.

The chosen antenna type for generating and receiving the beam were smooth-walled conical horns. The main advantages are their extremely high radiation efficiency achieving 98%, as well as a high coupling efficiency to gaussian beams of 91% (Eq. 11), allied to relatively easy manufacturing. Their parameters are detailed in Table 2 and schematically represented in Fig. 2a. Although corrugated horns are even better coupled to gaussian beams, their increased complexity make them more difficult to manufacture. Finally, parabolic reflectors were used, as they were donated for this project.

**Table 2:** Conical horn antenna design parameters.

Symbol	Terminology	Value (cm)
*a*	Aperture radius	10.893
$R_h$	Slant length	30.587
$L_h$	Horn length	28.582
$l_w$	Waveguide length	5.224
*G*	Gain	19 dB
$\varpi _0$	Beam waist	4.902
$z_0$	Beam waist location	8.719

Having defined the system’s fundamental blocks, the components were adjusted for a ${5.8}\,{\textrm{GHz}}$ microwave beam propagating a distance of ${5}\,{\textrm{m}}$. The choice of frequency was due to the state-of-the-art, while the distance was considered for being simultaneously feasible, challenging and comparable to William Brown’s record experiment of ${7.62}\,{\textrm{m}}$.

From this starting point, the parameter values that had to be chosen were the generated gaussian beam waist, $\varpi _{0_{gen}}$, the reflectors’ focal length, *f*, and the distance between both components, $d_{in}$. Since $d_{in}$ is adjustable at any time, the other two parameters were decided iteratively: for a reflector dish with a diameter of ${1}\,{\textrm{m}}$, the necessary gaussian beam would need to have $\varpi _0 = {4.902}\,{\textrm{cm}}$; the conical horn antenna final dimensions were determined after following the design principles found in^25^ and are detailed in Table 2. After their simulation and optimization using the CST Studio Suite software, they were 3D printed and covered with copper, as can be seen in Fig. 2b and c. Following their testing in an anechoic chamber, the $S_{11}$ parameters (Fig. 2d) were used to calculate the antennas’ gain by the substitution method. Furthermore, after the acquisition of reflectors with acceptable parameters, presented in Table 3, $d_{in}$ was adjusted for the intended transfer distance.

**Table 3:** Reflector parameters.

Symbol	Terminology	Value (cm)
*W*	Width	105.897
*f*	Focal length	72.675
$H_{phy}$	Physical height	114.531

**Table 4:** Complete WPT system parameter values.

Symbol	Terminology	Value (cm)
*L*	Transfer distance	${5}\,{\textrm{m}}$
$\omega$	Frequency of operation	${5.8}\,{\textrm{GHz}}$
*f*	Reflector’s focal length	72.675
$d_{in}$	Input beam distance	87.270
$\varpi _{0_{gen}}$	Generated beam waist	4.902
$\varpi _{0_{prop}}$	Propagating beam waist	17.254

**Figure 3:**
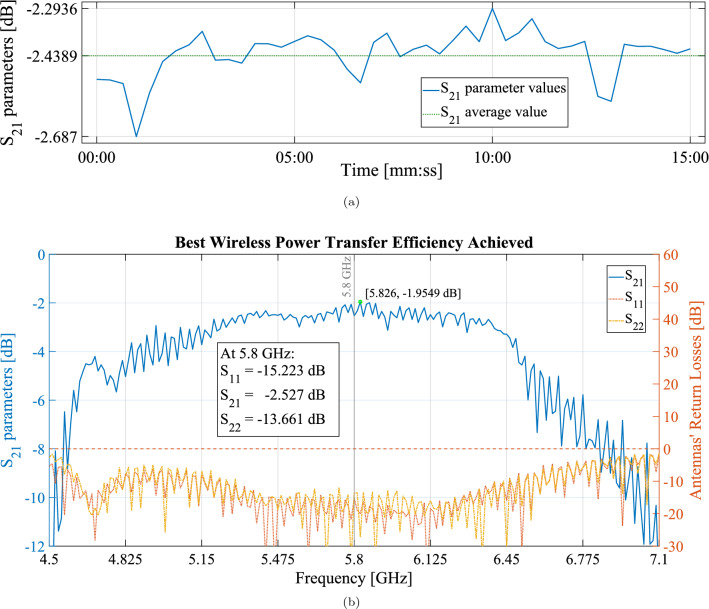
(**a**) Time sweep of the $S_{21}$ parameter at ${5.8}\,{\textrm{GHz}}$, taken every 20 s for 15 mins, showing a WPT efficiency over $53\%$ during the whole time. (**b**) All *S* parameters corresponding to the best efficiency achieved in this set of experiments: $S_{21}$ parameters on the left axis and the horn antennas’ return losses on the right one. A maximum beam efficiency of 63.75% was achieved.

Hence, the complete system parameter values are described in Table 4. These were validated in a script from which the schematic of the complete system, represented in Fig. 1b, was obtained.

From the theoretical analysis, a preliminary experiment was prepared for measuring the focus location, using only one of the parabolic reflectors. Following its validation, the complete system was set-up and experimented upon. Both the preliminary and the final results used a Keysight FieldFox Microwave Analyzer N9918A connected to the horn antennas by CBL-2M-SMSM+ cables, at a power level of ${0}\,{\textrm{dBm}}$.

The $S_{21}$ parameters were used as indicators of the beam efficiency. As mentioned previously, the microwave generation and reception losses were not accounted for since the network analyzer was used. Similarly, the cables’ losses were discarded due to the calibration performed everyday before the experiments took place. Therefore, the $S_{21}$ results provide information regarding the horn antennas’ and parabolic reflectors’ efficiency and, most importantly, the microwave beam collection efficiency throughout the system.

Finally, a calculation of the estimated efficiency was done: the horn antennas were designed in CST Studio Suite and an efficiency over $98\%$ was provided by the software. Since no practical measurements were done on the antennas’ efficiency, this value was used in the calculation. The reflectors’ efficiency was provided by the manufacturer as being $74\%$. Due to the system’s design always keeping the beam radius smaller than the components’ aperture, the spillover losses are negligible. Considering all this information, the maximum expected beam efficiency was $\eta _{Beam_{est.}} = 0.98^2 \times 0.74^2 = 52.6\%$.

## 5-meter wireless power transfer

A preliminary analysis was performed to verify that the radiation focus location was indeed at the expected distance from the reflector of *L*/2 = ${2.5}\,{\textrm{m}}$^23^. This verification consisted of measuring the $S_{21}$ parameters of both the horn antennas, while one served as the reflector’s feed and the other was used to sweep the distance from the reflector, at a height of 1 meter.

The maximum power received on this preliminary experiment was ${-12.537}\,{\textrm{dBm}}$ at a distance of ${245}\,{\textrm{cm}}$ from the reflector, a value close to the theoretical one of ${250}\,{\textrm{cm}}$. The main reasons for the displacement are attributed to possible system misalignments or to a difference in the gaussian beam parameter of the generated antenna. Either way, these are minimum and were accounted for in the complete system experiment.

**Figure 4:**
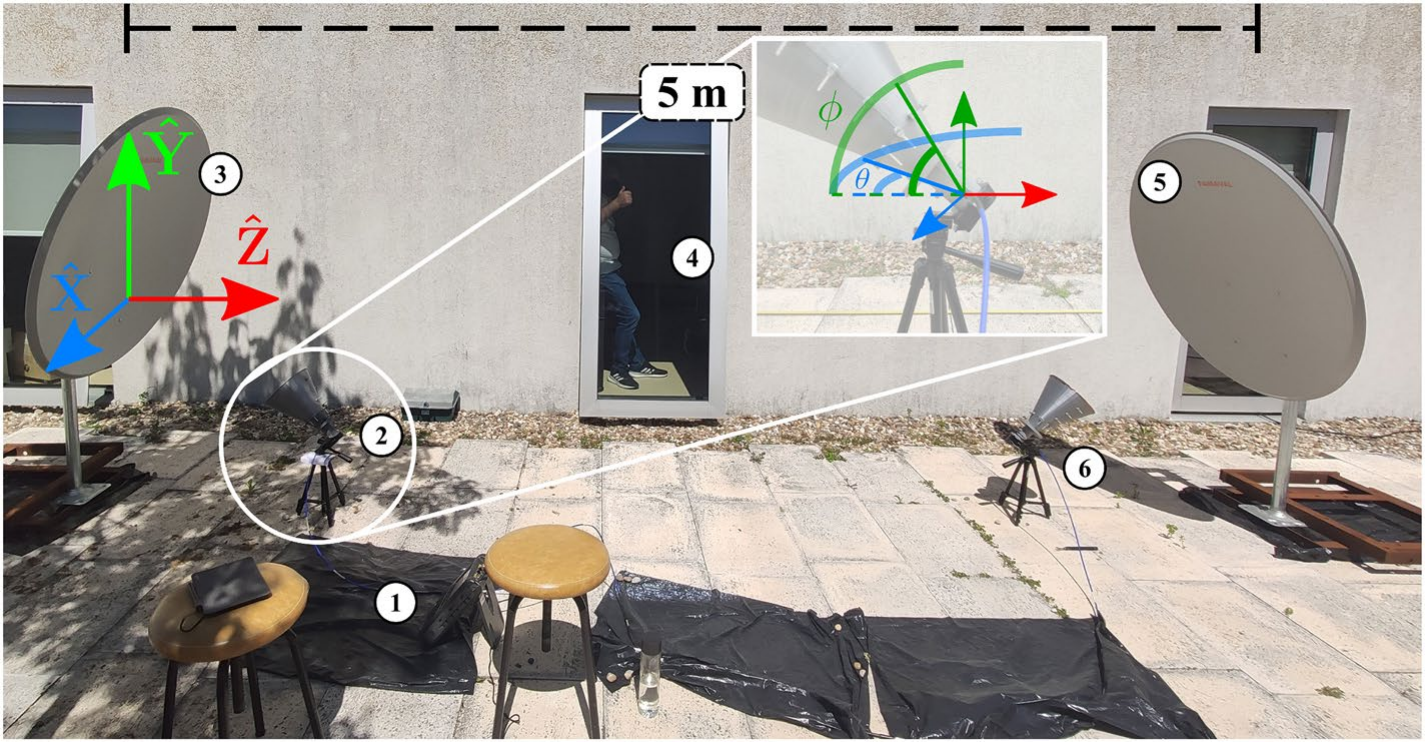
Complete ${5}\,{\textrm{m}}$ double-reflector system overview with position axis and attitude angles yaw, $\theta$, and pitch, $\phi$. The components are: (1) Microwave generator and receiver; (2) Transmitting horn antenna; (3) First parabolic reflector; (4) Beam focus location; (5) Second parabolic reflector; (6) Receiving horn antenna.

These preliminary results validated the theoretical analysis, prompting the complete system set-up for the ${5}\,{\textrm{m}}$ WPT experiment. Due to the size of the complete system, the experiments had to take place on the outside patio of the lab facilities, as can be seen in Fig. 4. In this figure, all the systems’ components are presented, as well as the axis of reference. Following the power flow, the components are the microwave generator and receiver (1), the transmitting horn antenna (2), the first parabolic reflector, responsible for focusing the beam at ${2.5}\,{\textrm{m}}$ (3), the beam focus location (4), the second reflector, responsible for transforming the beam for optimal reception (4) and finally the receiving horn antenna (5).

With these components, two different sets of measurements were conducted: the first consisted in discovering the optimal parameter values and measuring the efficiency value for 15 minutes, while the latter consisted of a thorough parameter variation for observing their effect on the overall WPT efficiency.

Starting from the theoretically optimal values, various parameters were adjusted in order to arrive at the maximum WPT efficiency.

The complete parameters to be optimized were both the horn antennas’ position and attitude and the parabolic reflectors’ attitude. Each of these is a product of multiple components, totaling in 14 parameters to explore: each antenna had three positioning axis, xyz, and two angles, yaw ($\theta$) and pitch ($\phi$); the reflector’s parameters possible of adjustment were only its attitude angles, elevation and horizontal sweep. These parameters are visibly explained in Fig. 4. The horn antennas’ rolling angle was not varied since it is related to the their linear polarization. Therefore, the best case was when their roll angle was the same or at a 180 degree rotation.

Finally, a 15 minutes experiment was conducted at the optimal point, with measurements being taken every 20 seconds. In this manner, it was possible to achieve a power transfer efficiency over 53$\%$, as seen in Fig. 3a. The total 45 points averaged at ${-2.439}\,{\textrm{dB}} (57.0\%)$ for which one can conclude that ${0.513}\,{\textrm{J}}$ were transferred during this time.

The following conclusions were made possible: although the measurements were taken in an outdoors location, the wind effects were usually not significant. However, on rare occasions with stronger wind intensity, the systems’ components were redirected to the point where a difference in the beam efficiency was noted. Nevertheless, when the intensity reduced again, the efficiency would return to the previous nominal values, indicating that the components’ fixation mechanism was not perfect but suitable for the experiment. Frequently, this phenomenon turned out to be advantageous because efficiency values better than thought to be achievable with the current system would be measured, prompting further alignment efforts.

In an additional note, at the frequency of operation of 5.8 GHz, the atmospheric humidity does not affect the microwave transmission efficiency, as can be obtained from the model presented in^26^. We can conclude that the beam efficiency does not depend on it, which is one of the reasons why microwaves are adequate for WPT through the Earth’s atmosphere.

## System sensitivity

The other set of measurements consisted in observing how the beam transfer efficiency varied with the different parameters and quantify the system’s sensitivity. Therefore, from the optimal point obtained previously, a thorough variation of the most important variables was performed.

Attentively, in order to reduce the wind’s impact in the measurements, the following results were manually repeated five times for each configuration, with the average being taken as the most indicative value.

**Figure 5:**
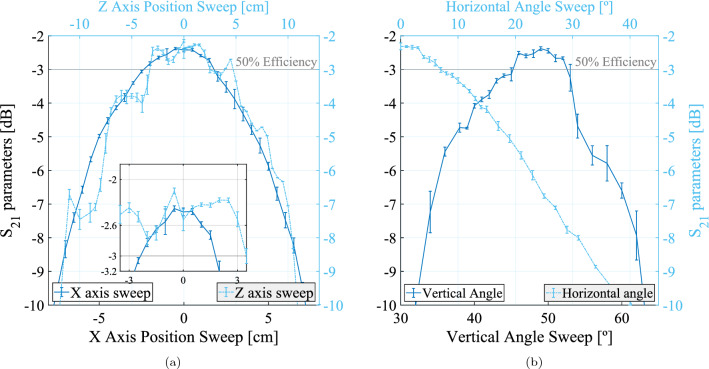
(**a**) Positioning parameters’ influence on the efficiency: transmitting horn antenna’s positioning in the $\hat{\text {Z}}$ axis and receiving horn antenna’s positioning in the $\hat{\text {X}}$ axis. (**b**) Attitude parameters’ influence on the efficiency: transmitting horn antenna’s yaw, $\theta$, and pitch, $\theta$, angles.

The parameters that were varied in this analysis were the transmitting horn antenna’s positioning in the $\hat{\text {Z}}$ axis and the receiving horn antenna’s positioning in the $\hat{\text {X}}$ axis, whose results are summarized in Fig. 5a, as well as the transmitting antenna’s yaw, $\theta$, and pitch, $\theta$, angles, presented in Fig. 5b. From them, it is possible to conclude that the system is highly sensitive, with the vertical angle being the most sensitive of all parameters (Table 5). These results are important for setting up future experiments: the components’ attitude must be explored first, since they are more impactful in the overall system alignment.

**Table 5:** System sensitivity summary, at ${5.8}\,{\textrm{GHz}}$.

Parameter		Unit	Efficiency reduction at	
			− 5 [unit]	5 [unit]
Position	Z Axis	cm	31.374%	24.039%
	X Axis		53.109%	48.481%
Angle	Vert.	deg.	16.894%	41.153%
	Horiz.		/	8.6139%

The efficiency reduction for each parameter is a comparison between the maximum value and that at the specified variation, for each parameter sweep.

It is also possible to observe that the received gaussian beam’s waist can be determined from the $\hat{\text {X}}$ axis variation. Its electric power distribution falls to 1/*e* at around ${-6}$ and ${5.5}\,{\textrm{cm}}$, hence, the beam waist can be calculated as ${5.75}\,{\textrm{cm}}$ using this method, approximate to the theoretical value of ${4.90}\,{\textrm{cm}}$.

Finally, this thorough parameter variation enabled the measurement of the best wireless power transfer efficiency achieved, verified to be ${63.75}{\%}$, as is visible in Fig. 3b.

## Figure-of-merit relating physical dimensions

Aiming to compare our results with the SOA, but lacking to find a figure-of-merit (FOM) in the literature, we propose new ones. Their purpose is to facilitate the comparison between WPT projects in terms of the systems’ physical dimensions, by giving a fast and easily calculable quantity, relating the *power transfer distance* (*L*) with the *components' largest dimension* (*D*), the number of components (*n*) used besides the transmitting and receiving antennas, and the *wavelength* $\lambda$. To compare the transfer distance to the other dimensional quantities, one can express their ratio.

Starting by comparing the transfer distance to the wavelength, we get $L / \lambda$, whose physical meaning is related to the beam’s divergence: for a fixed system, and therefore transfer distance, increasing the frequency of operation reduces the wavelength, resulting in an increase of the confocal distance (Eq. (10)), effectively reducing the beam’s divergence.

Then, we wanted to account for the various components and their sizes. The approach proposed here was inspired by the calculation of antennas’ field regions, where the antenna’s largest dimension, $D_{Ant.}$, is used^27^. Since we are analyzing complete systems, we calculate the ratio between the transfer distance and the sum of the largest dimension of all components. For fundamental WPT systems composed simply of the transmitting and receiving antennas, and assuming that they are equal, we get $L / (2 D_{Ant.})$. However, for complex systems with additional components, it would be cumbersome to account for all of them individually. We propose to take the largest dimension of all the system components and simply calculate the ratio $L / [(n + 2) D]$, where *n* is the number of components used besides the essential transmitting and receiving antennas. The latter were kept separate because they will always be necessary, which should help users account for all components.

Finally, both of these are multiplied together and converted to logarithmic scale, resulting in the *dimensional figure-of-merit*, $FOM_{Dim.}$:

1 $$\begin{aligned} FOM_{Dim.} = 10 \log \left[ \frac{L^2}{(n + 2) D \lambda } \right] . \end{aligned}$$

Note that this definition is generalized so that it can be applied even in WPT projects that do not use the quasioptics analysis.

Additionally, in order to have a single figure-of-merit capable of providing an overall idea of the beam subsystem of any WPT system, we also propose the following quantities that include the beam efficiency, $\eta _{Beam}$, as defined in Eq. (5):

2 $$\begin{aligned} FOM_{Beam_1} = 10 \log \left[ \frac{L^2}{(n + 2) D \lambda } \times \eta _{Beam} \right] \end{aligned}$$

and

3 $$\begin{aligned} FOM_{Beam_2} = 10 \log \left[ \frac{L^2}{(n + 2) D \lambda } \right] \times \eta _{Beam}, \end{aligned}$$

where the only difference between them is whether the beam efficiency is included in the logarithmic function or not. By having it separately, more weight is given to the beam efficiency, reason for which we expect it to be the preferred FOM.

All of these are applied to the state-of-the-art and compared in Table 1, where the system with the highest dimensional FOM is the work by Brown that transferred power through 1540 km^9^. Our solution is in third place, behind Brown’s and the work of Gonzalez^21^, which achieved a relatively high distance of power transfer for the high frequency of operation, although it is only 13.6 cm. On the other hand, for the 2^nd^ FOM that includes the beam efficiency and in which it is more impactful, Eq. (3), our work ranks highest for long-range applications, having achieved a high beam efficiency when considering the system components and power transfer distance.

## Discussion

The experimental results enabled the measurement of a beam efficiency higher than initially estimated. We believe this is due to the reflectors having a higher efficiency than provided by the manufacturers. Although the case can be made that this increase is due to reflections by the floor and walls, the high directivity of the beam discredits this possibility. The beam was intentionally blocked in multiple locations between both reflectors and its efficiency drop was only noticed in the central axis, as expected, meaning that the beam’s side lobes and ground or wall reflections are negligible.

As such, an overview of each components’ efficiency and their comparison with the estimated values are shown in Table 6.

**Table 6:** All components’ efficiency and overall beam efficiency, estimated and measured.

	$\eta _{Tx_{Ant.}}$	$\eta _{Coll.}$	$\eta _{Focus}$	$\eta _{Rx_{Ant.}}$	$\eta _{Beam}$
Est.	0.98	1	$0.740^2$	0.98	52.59%
Meas.	0.98	1	$0.815^2$	0.98	63.75%

In order to better quantify the system, the complete distance that the beam propagates is $L + 2 \times d_{in} = 5 + 2 \times 0.87270 = {6.745}\,{\textrm{m}}$. On the other hand, from a traditional antenna theory point of view, the transmitting and receiving blocks, each composed of a horn and a reflector, have a height of ${1.306}\,{\textrm{m}}$. All of this for a wavelength of ${0.0517}\,{\textrm{m}}$, which means that the distance between the reflectors is $\approx {97}{\lambda }$.

When comparing with the state-of-the-art in Table 1, the advantages of using quasioptics in WPT become clear: the spillover losses are negligible making this system the best beam efficiency ever designed for WPT, surpassing the previous works when taking into consideration the number of focusing components and transfer distance.

However, this system can be improved: the reflectors used were commercially available for telecommunication purposes, hence, the beam efficiency can be further improved by developing higher efficiency focusing elements. Additionally, the beam efficiency can be increased by using corrugated horns instead of the smooth-walled horns that were used since these antennas have a higher gaussian beam coupling.

## Conclusions

A complete 5-meter double-reflector system was experimented upon with the goal of achieving the highest wireless power transfer efficiency possible at ${5.8}\,{\textrm{GHz}}$, validating the theoretical analysis performed previously using quasioptics.

From this experiment, an efficiency of $63.75\%$ was obtained, showing that a microwave beam was successfully optimized for the system in question, enabling the significant reduction of spillover losses, to the point of them being negligible. The major losses were attributed to the parabolic reflectors used, showing that the development of higher efficiency focusing components is an important part of the future of wireless power transfer.

This is because the losses added by the focusing components for controlling the beam’s divergence, are offset by a reduction in the spillover losses, enabling an overall increase in beam efficiency.

Furthermore, the dependence of the beam transfer efficiency with different system parameters has been discussed, as well as the wind’s impact on the experiment. The receiving gaussian beam waist was also measured to be ${5.75}\,{\textrm{cm}}$.

On the other hand, several figures-of-merit have been proposed, aiming to compare various WPT systems with the power transfer distance and beam efficiency, advancing the state-of-the-art in WPT analysis.

Following this work, the same double-reflector system can be used for further validating the quasioptical usage in WPT systems. The system can be optimized for longer distances by adjusting some system parameters.

On the other hand, the eventual usage of a rectenna for receiving power should reduce the system’s sensitivity, albeit with a possible loss in the receiving antenna efficiency. Finally, the power generation can also be implemented so that a complete WPT efficiency can be measured.

On a final note, although the principles of quasioptics have been developed for some decades, these have been mostly applied for microwave astronomy and to study materials. The development of a complete system with low spillover losses for wireless power transfer has not been proposed until now, nor experimented upon.

The positive results obtained here are evidence that the quasioptical framework is advantageous for developing radiative wireless power transfer systems with high efficiency.

## Methods

### Beam efficiency

It is worth noting that the beam efficiency, $\eta _{Beam}$, defined in this study may differ from the literature: here it consists of all the EM radiation components except its generation, $\eta _{DC-RF}$, and rectification, $\eta _{DC-RF}$. Hence, any radiative WPT system is divided in the following components:

4 $$\begin{aligned} \eta _{WPT} = \eta _{DC-RF} \ \eta _{Beam} \ \eta _{RF-DC}, \end{aligned}$$

where

5 $$\begin{aligned} \eta _{Beam} = \eta _{Tx_{Ant.}} \ \eta _{Coll.} \ \eta _{Focus} \ \eta _{Rx_{Ant.}}. \end{aligned}$$

This definition was chosen for it encompasses all the microwave phenomena in one efficiency parameter. Furthermore, all the components developed in the present work have their efficiencies included in the beam’s efficiency, namely the transmitting and receiving horn antennas, $\eta _{Tx_{Ant.}}$ and $\eta _{Rx_{Ant.}}$, as well as the focusing components, $\eta _{Focus}$. The final parameter, $\eta _{Coll.}$, is the beam collection efficiency throughout the complete system, which is associated with the spillover losses and system components’ alignment. An important discussion on the beam collection efficiency calculation is presented in^28^ where a new method is proposed and compared with the typical Friis’ and Goubau’s formulas.

### Quasioptics

Assuming a propagation in the ${\hat{z}}$ direction, $z_0$ is the point at which the power is most concentrated and the divergence less evident. The electric field of a gaussian beam that propagates freely in the fundamental mode is axially symmetric, depending only on the distance from the axis of propagation (radius), *r*, and the position along the axis, *z*:

6 $$\begin{aligned} E(r,z) = \sqrt{ \frac{2}{ \pi \varpi ^2} } \exp \left( - \frac{r^2}{\varpi ^2} - ikz - \frac{i \pi r^2}{ \lambda R} + i \phi _0 \right) , \end{aligned}$$

where $\varpi$ is the beam radius, *R* is the radius of curvature of the wave front, $\phi _0$ is the phase shift and $\lambda$ is the wavelength.

The beam radius is the most relevant quantity for WPT, since it is defined as the radial distance at which the power density falls to 1/*e* of the on-axis value, with *e* being the Euler’s number. Its minimum value is a characteristic of the beam, called the beam waist, $\varpi _0$, located in $z_0$.

The basics of beam transformation will now be introduced: any QO system can be represented by a matrix, $M_{sys}$,

7 $$\begin{aligned} M_{sys} = \begin{bmatrix} 1 &{} d_{out} \\ 0 &{} 1 \end{bmatrix} \cdot \begin{bmatrix} A &{} B \\ C &{} D \end{bmatrix} \cdot \begin{bmatrix} 1 &{} d_{in} \\ 0 &{} 1 \end{bmatrix} \end{aligned}$$

and its effect on the microwave beam can be understood by the relationship between the input and output beam waist and distance to the system, $\varpi _{0_{in}}$, $d_{in}$ and $\varpi _{0_{out}}$, $d_{out}$, respectively:

8 $$\begin{aligned} d_{out} = - \frac{(Ad_{in} + B)(Cd_{in} + D) + AC z_c^2}{{(Cd_{in} + D)^2 + C^2 z_c^2}} \end{aligned}$$

and

9 $$\begin{aligned} \varpi _{0_{out}} = \frac{ \varpi _{0_{in}}}{{\sqrt{(Cd_{in} + D)^2 + C^2 z_c^2}}}, \end{aligned}$$

where $z_c$ is the *confocal distance*, a parameter which details the distance from $z_0$ where the beam remains collimated, presenting minimum divergence:

10 $$\begin{aligned} z_c = \frac{\pi \varpi _0^2}{\lambda }. \end{aligned}$$

Finally, for generating quasioptical beams, a comparison between its electric field and that of any antenna’s radiation quantifies how approximate both are through the coupling efficiency, $\eta _G$,^20,29^:

11 $$\begin{aligned} \eta _G = \frac{{\iint \left| E_A \cdot E_G^* \right| ^2 dx \, dy }}{{ \left[ \iint |E_A|^2 dx \, dy \right] \left[ \iint |E_G|^2 dx \, dy \right] }} \end{aligned}$$

Reference^29^ presents an algorithm that outputs the fundamental gaussian beam parameters, $\varpi _0$ and $z_0$, maximizing $\eta _G$ for any input antenna’s far-field radiation pattern.

### Gaussian beam of conical horn antennas

The QO analysis of smooth-walled conical horn antennas showed that they achieve one of the highest coupling efficiencies with $\eta _G=91\%$^20^. The relationship between the gaussian beam radius at the antenna’s aperture and the aperture radius, *a*, is given by $\varpi = c_g a$, where $c_g = 0.76$. The beam waist and its location can be obtained from (12) and (13) by taking the gaussian beam’s radius of curvature as the antenna’s slant length, $R = R_h$^20^:

12 $$\begin{aligned} \varpi _0 = \frac{\varpi }{\left[ 1 + \left( \pi \varpi ^2 / \lambda R \right) ^2 \right] ^{0.5}} \end{aligned}$$

and

13 $$\begin{aligned} z_0 = \frac{R}{1 + \left( \lambda R / \pi \varpi ^2 \right) ^2}. \end{aligned}$$

### Double-reflector quasioptical analysis

The main results of the double-reflector system theoretical analysis^22^, are the relationships between the reflectors’ *focal length*, *f*, and the wireless power *transfer distance*, *L*:

14 $$\begin{aligned} f = \frac{z_c^2 + d_{in}^2}{z_c + d_{in}} \qquad \text {and} \qquad L = z_c + \frac{d_{in}^2}{z_c}. \end{aligned}$$

## Data Availability

The datasets used and analysed during the current study are available from the corresponding author on reasonable request.
